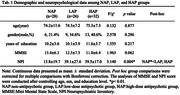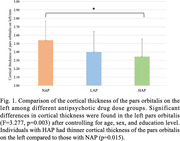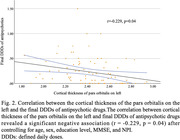# Exploring the Relationship Between Brain Structural and Antipsychotic Drug Dosage in BPSD

**DOI:** 10.1002/alz.087912

**Published:** 2025-01-03

**Authors:** Bo Hong, Tianli Tao, Han Zhang, Xia Li, Jianhua Chen, Ling Yue

**Affiliations:** ^1^ Shanghai Mental Health Center, Shanghai Jiao Tong University School of Medicine, Shanghai China; ^2^ Alzheimer’s Disease and Related Disorders Center, Shanghai Jiao Tong University, Shanghai China; ^3^ School of Biomedical Engineering, ShanghaiTech University, Shanghai China

## Abstract

**Background:**

Psychotropic medications are commonly prescribed for behavioral and psychological symptoms of dementia (BPSD). However, the safety of antipsychotic drugs has always been a concern. The study aims to investigate the relationship between brain structural and the effective dosage of antipsychotic drugs.

**Method:**

All the subjects were from Shanghai Mental Health Center. On baseline, the BPSD patients completed a 3D T1 MRI scan and evaluated by MMSE and Neuropsychiatric Inventory (NPI). Patients who underwent standardized clinical treatment were followed up until BPSD remission. Drug treatment procedures were recorded, with the antipsychotic drugs being converted using a defined daily doses (DDDs) method to obtain the final daily doses. We divided the patients into three groups: DDDs = 0, 0<DDDs<0.3, and DDDs≥0.3, representing a non‐antipsychotic group (NAP), a low‐dose group (LAP), and a high‐dose group (HAP). The relationship between the altered brain regions and the DDDs were investigated.

**Result:**

A total of 86 BPSD patients were enrolled (NAP = 28, LAP = 26, HAP = 32). Among the three groups, NAP group showed less NPI score, while no difference was observed in age, gender, education level, and MMSE (Tab. 1). ANCOVA analysis showed significant differences in the cortical thickness of pars orbitalis on the left (F = 3.277, p = 0.003) and the volume of thalamus on the left (F = 4.279, p<0.001) among three groups. *Post hoc* analysis indicated that HAP group had thinner cortex in the left pars orbitalis compared to NAP group (Fig. 1). Ordinal logistic regression analysis revealed that NPI (p = 0.014) and cortical thickness at the left pars orbitalis (p = 0.037) were independent predictors of DDDs. Further association analysis revealed a significant negative correlation between cortical thickness of left pars orbitalis and DDDs (r = ‐0.229, p = 0.04), even after adjusting for gender, age, education, MMSE, and NPI. (Fig. 2).

**Conclusion:**

This study provides the first‐ever evidence that brain anatomical changes may serve as valuable biomarkers in the prediction of antipsychotic drug dosage for BPSD. The result has significant implications for optimizing clinical management strategies and offers insights into the intricate neuropathological mechanisms of BPSD.